# Supplementary material for: Co-Targeting of DTYMK and PARP1 as a Potential Therapeutic Approach in Uveal Melanoma
Source: Cells. 2024 Aug 14;13(16):1348. doi: 10.3390/cells13161348 (PMC11352547; doi:10.3390/cells13161348)
Supplement: Supplementary file 1 [file cells-13-01348-s001.zip › cells-3106462-supplementary.pptx]

## Slide 1
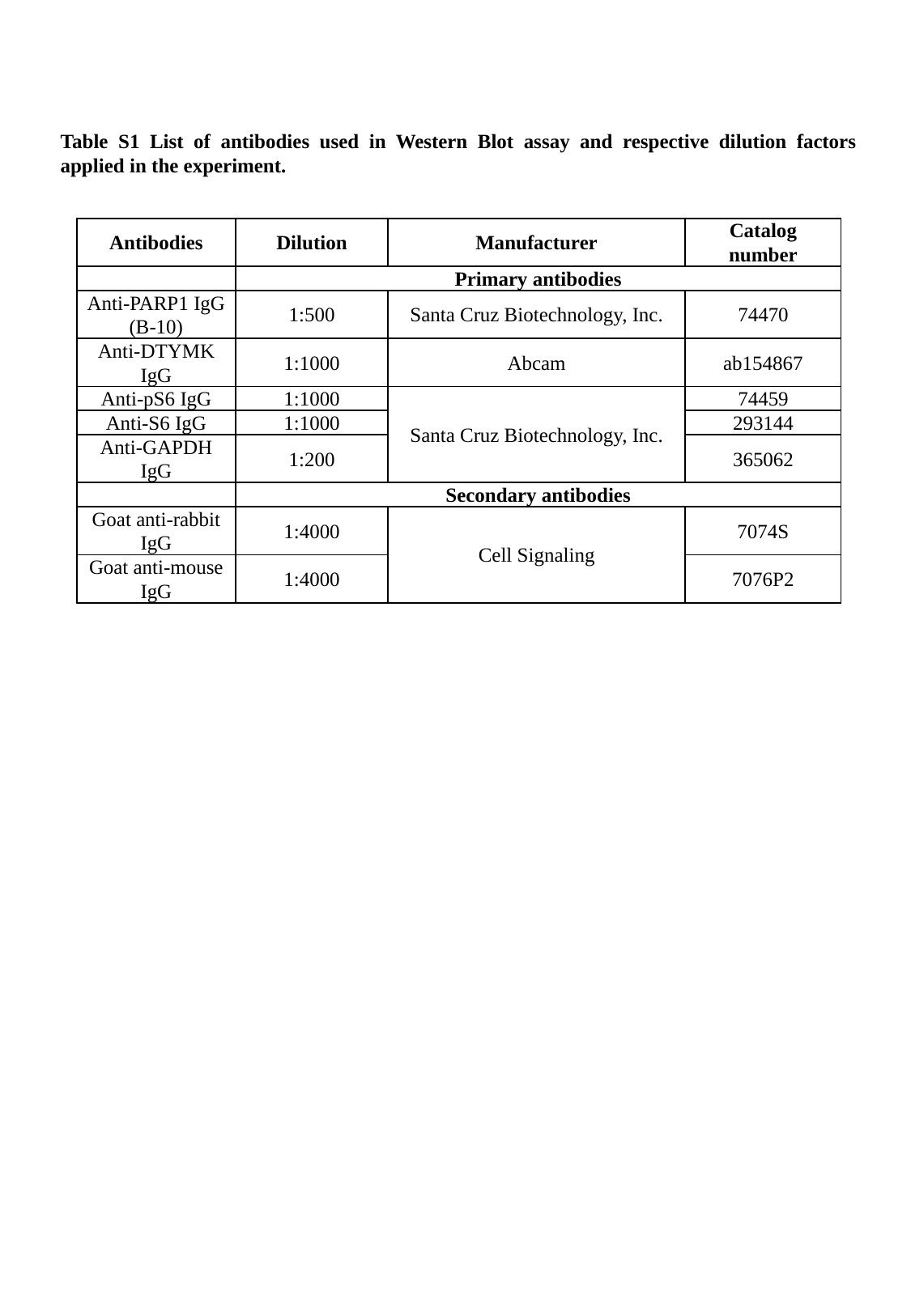

Table S1 List of antibodies used in Western Blot assay and respective dilution factors applied in the experiment.
| Antibodies | Dilution | Manufacturer | Catalog number |
| --- | --- | --- | --- |
| | Primary antibodies | | |
| Anti-PARP1 IgG (B-10) | 1:500 | Santa Cruz Biotechnology, Inc. | 74470 |
| Anti-DTYMK IgG | 1:1000 | Abcam | ab154867 |
| Anti-pS6 IgG | 1:1000 | Santa Cruz Biotechnology, Inc. | 74459 |
| Anti-S6 IgG | 1:1000 | | 293144 |
| Anti-GAPDH IgG | 1:200 | | 365062 |
| | Secondary antibodies | | |
| Goat anti-rabbit IgG | 1:4000 | Cell Signaling | 7074S |
| Goat anti-mouse IgG | 1:4000 | | 7076P2 |

## Slide 2
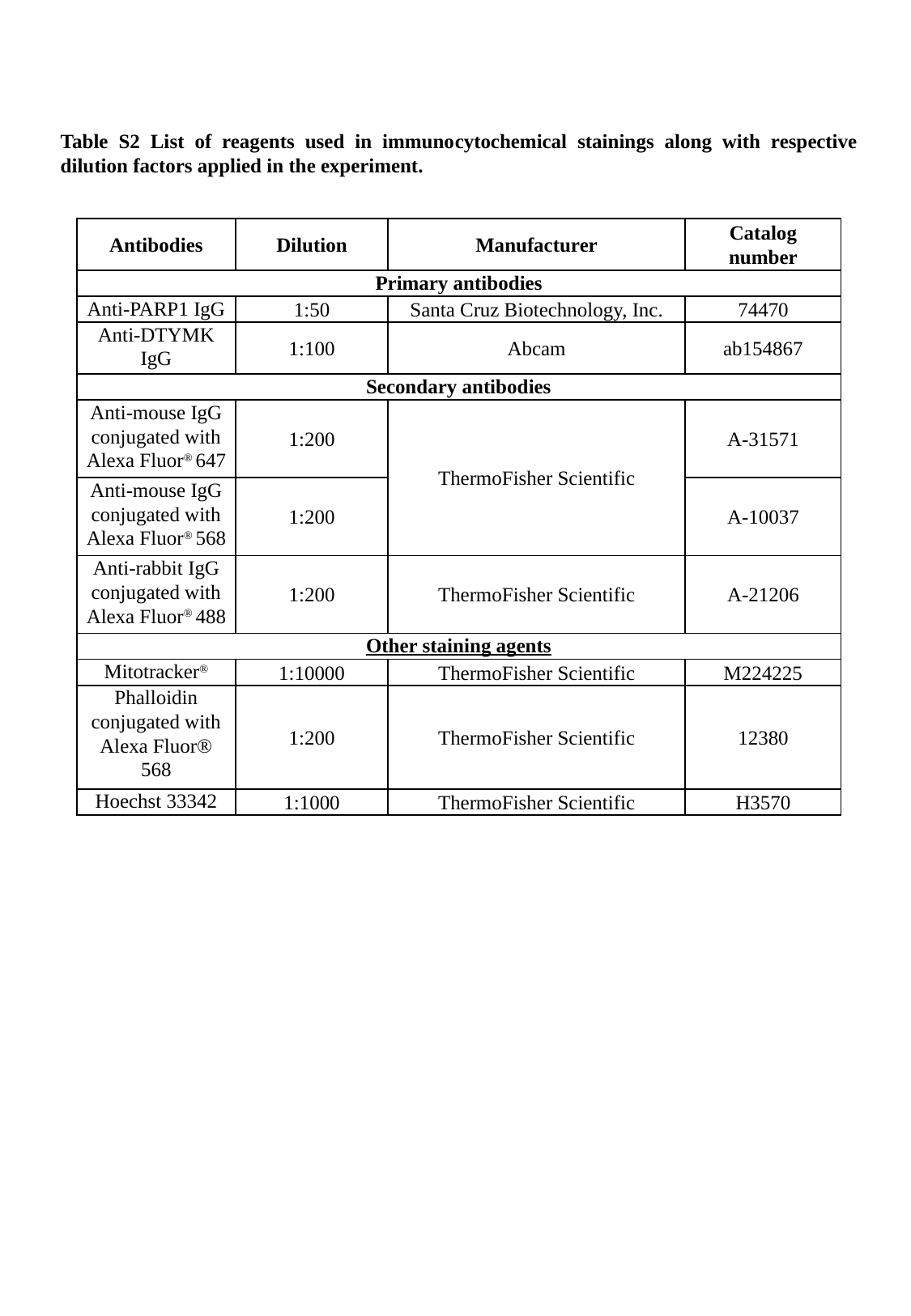

Table S2 List of reagents used in immunocytochemical stainings along with respective dilution factors applied in the experiment.
| Antibodies | Dilution | Manufacturer | Catalog number |
| --- | --- | --- | --- |
| Primary antibodies | | | |
| Anti-PARP1 IgG | 1:50 | Santa Cruz Biotechnology, Inc. | 74470 |
| Anti-DTYMK IgG | 1:100 | Abcam | ab154867 |
| Secondary antibodies | | | |
| Anti-mouse IgG conjugated with Alexa Fluor® 647 | 1:200 | ThermoFisher Scientific | A-31571 |
| Anti-mouse IgG conjugated with Alexa Fluor® 568 | 1:200 | | A-10037 |
| Anti-rabbit IgG conjugated with Alexa Fluor® 488 | 1:200 | ThermoFisher Scientific | A-21206 |
| Other staining agents | | | |
| Mitotracker® | 1:10000 | ThermoFisher Scientific | M224225 |
| Phalloidin conjugated with Alexa Fluor® 568 | 1:200 | ThermoFisher Scientific | 12380 |
| Hoechst 33342 | 1:1000 | ThermoFisher Scientific | H3570 |

## Slide 3
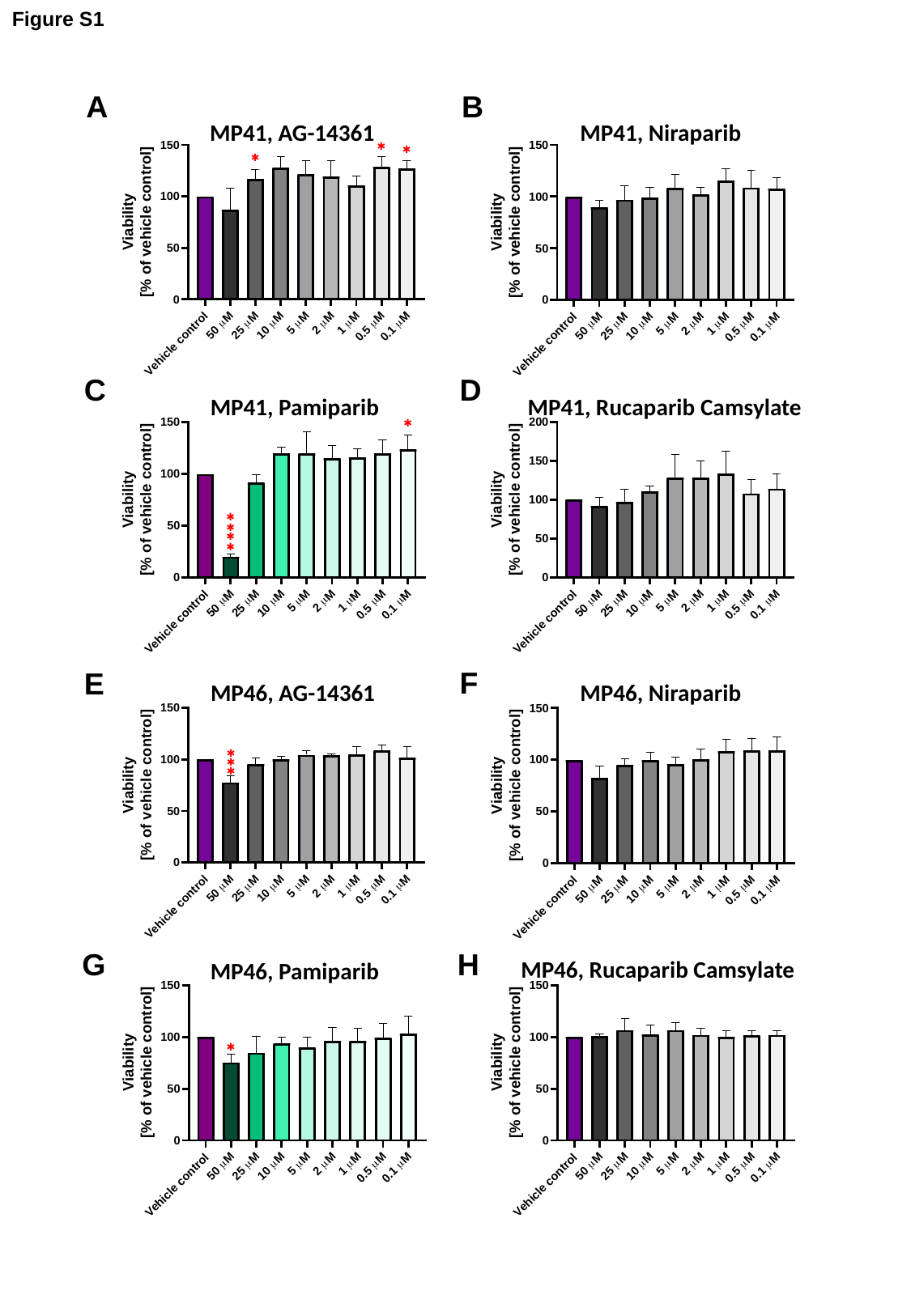

Figure S1
B
A
MP41, AG-14361
MP41, Niraparib
D
C
MP41, Pamiparib
MP41, Rucaparib Camsylate
F
E
MP46, AG-14361
MP46, Niraparib
H
G
MP46, Rucaparib Camsylate
MP46, Pamiparib

## Slide 4
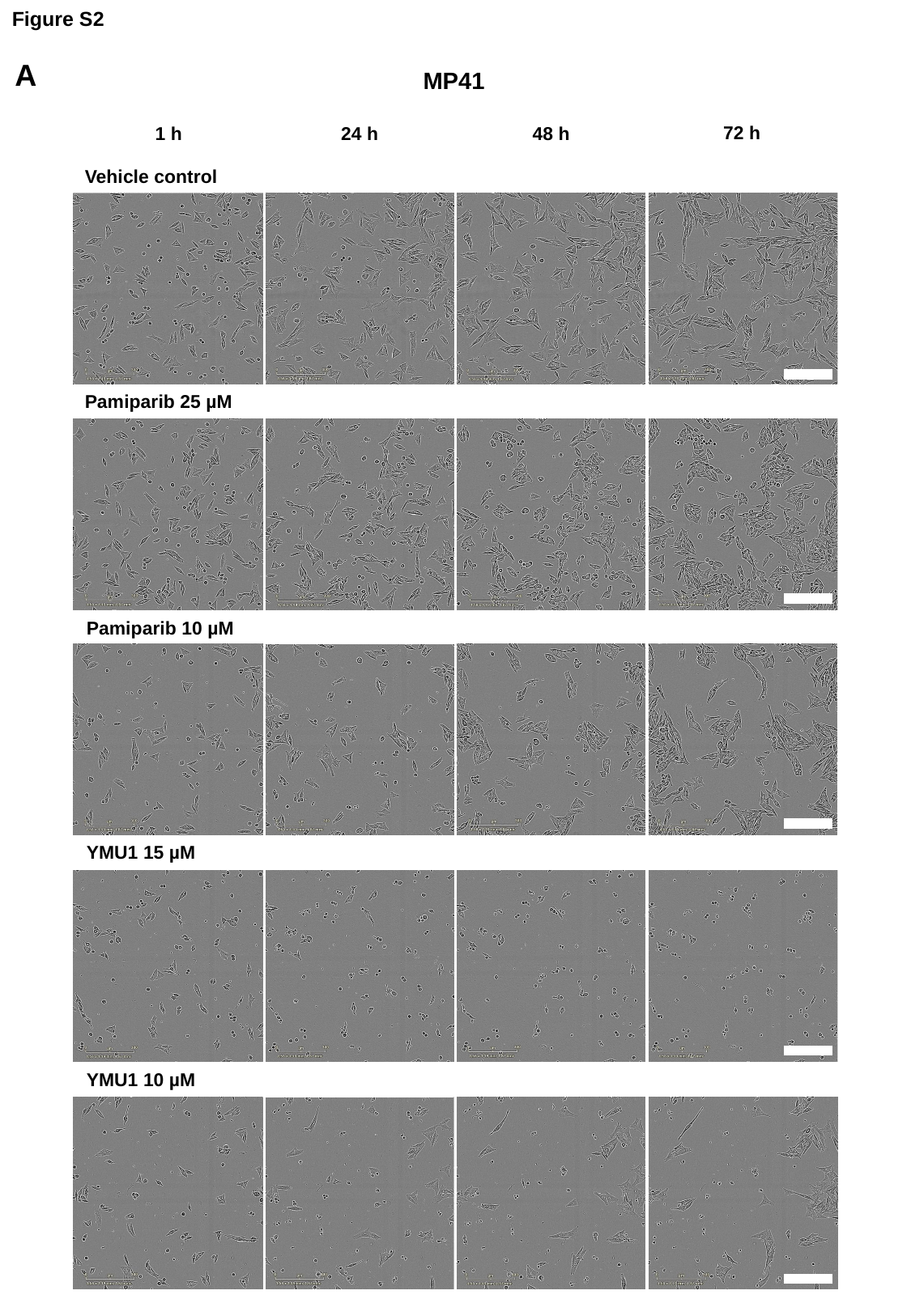

Figure S2
A
MP41
72 h
1 h
24 h
48 h
Vehicle control
Pamiparib 25 µM
Pamiparib 10 µM
YMU1 15 µM
YMU1 10 µM

## Slide 5
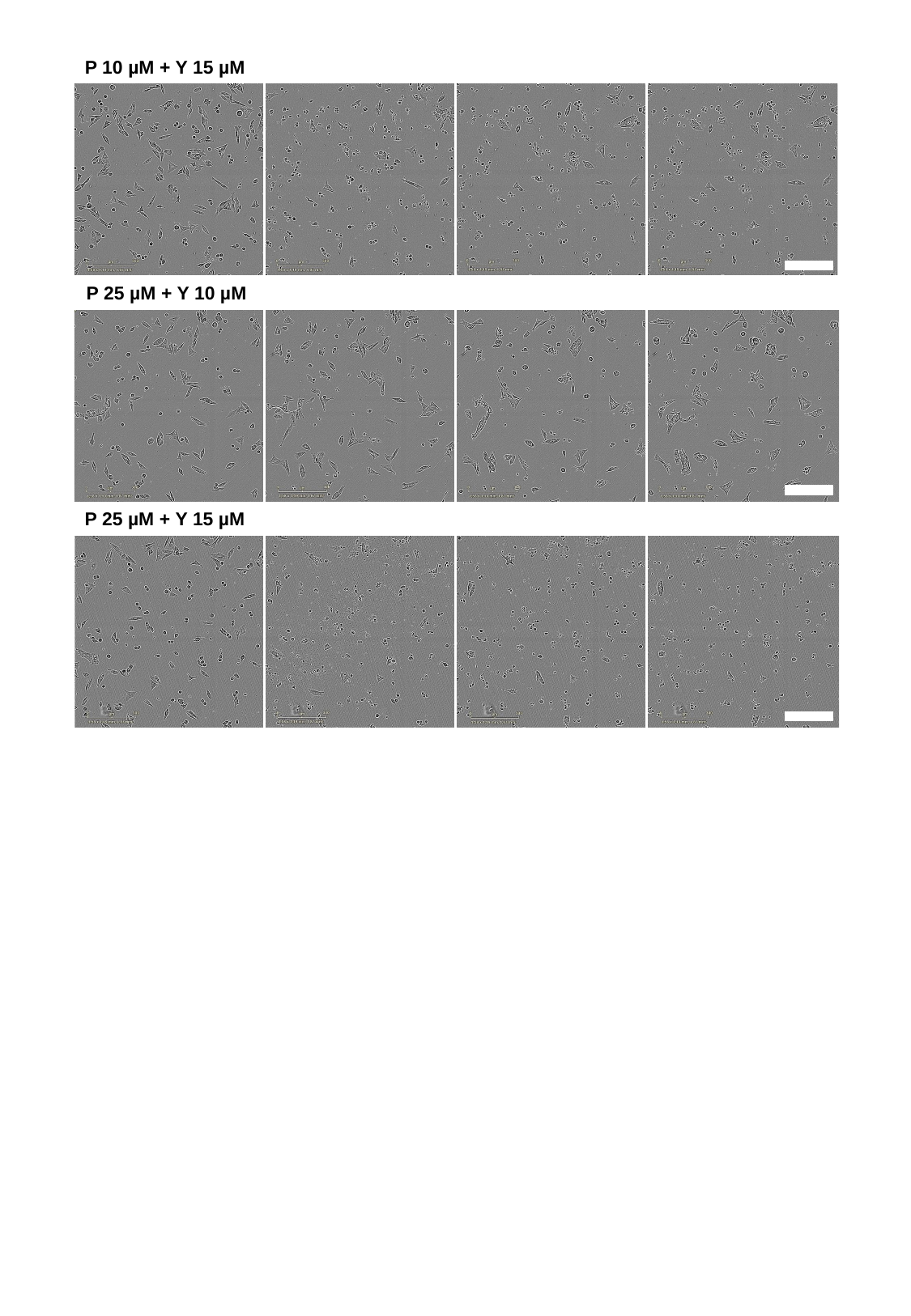

P 10 µM + Y 15 µM
P 25 µM + Y 10 µM
P 25 µM + Y 15 µM

## Slide 6
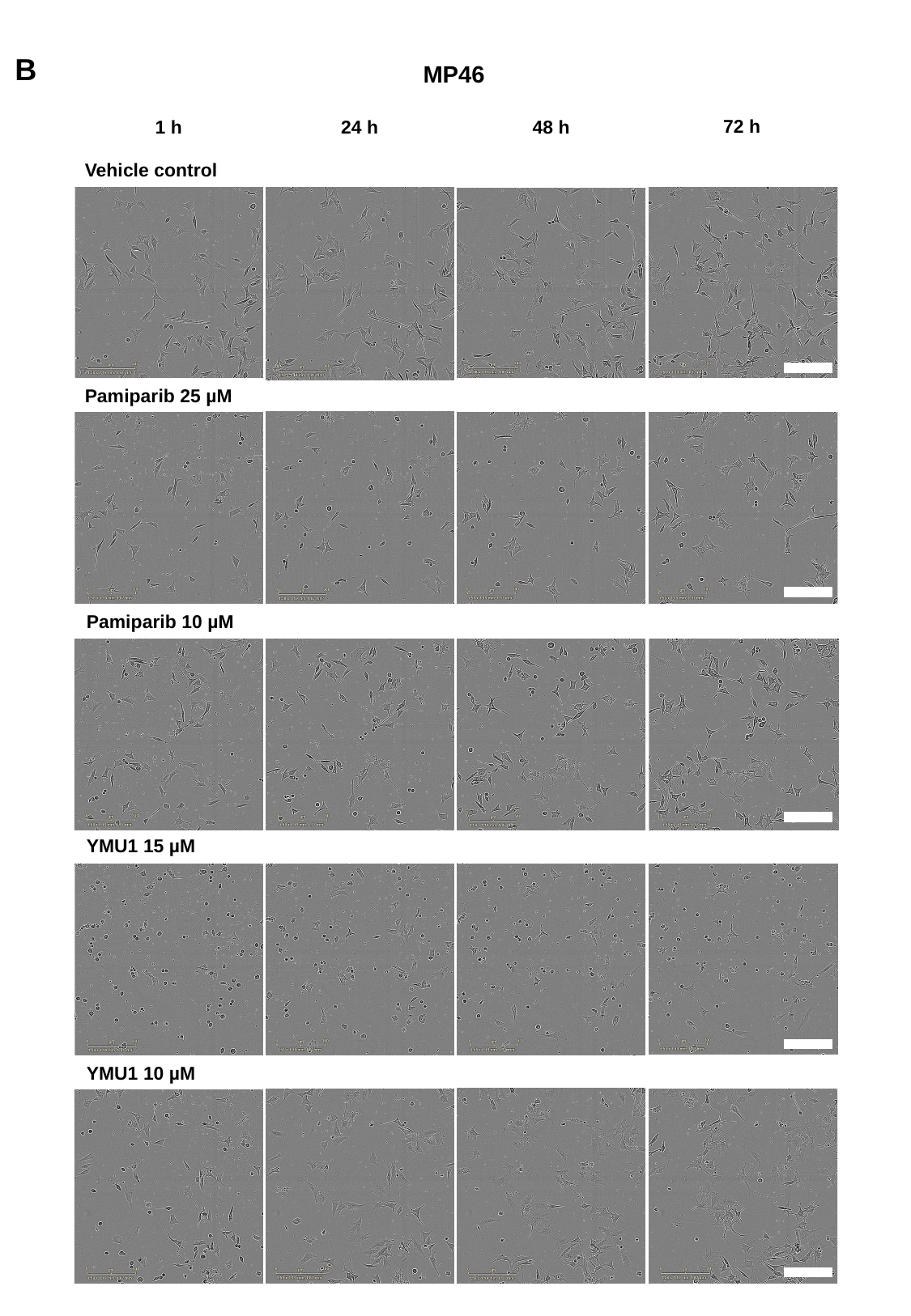

B
MP46
72 h
1 h
24 h
48 h
Vehicle control
Pamiparib 25 µM
Pamiparib 10 µM
YMU1 15 µM
YMU1 10 µM

## Slide 7
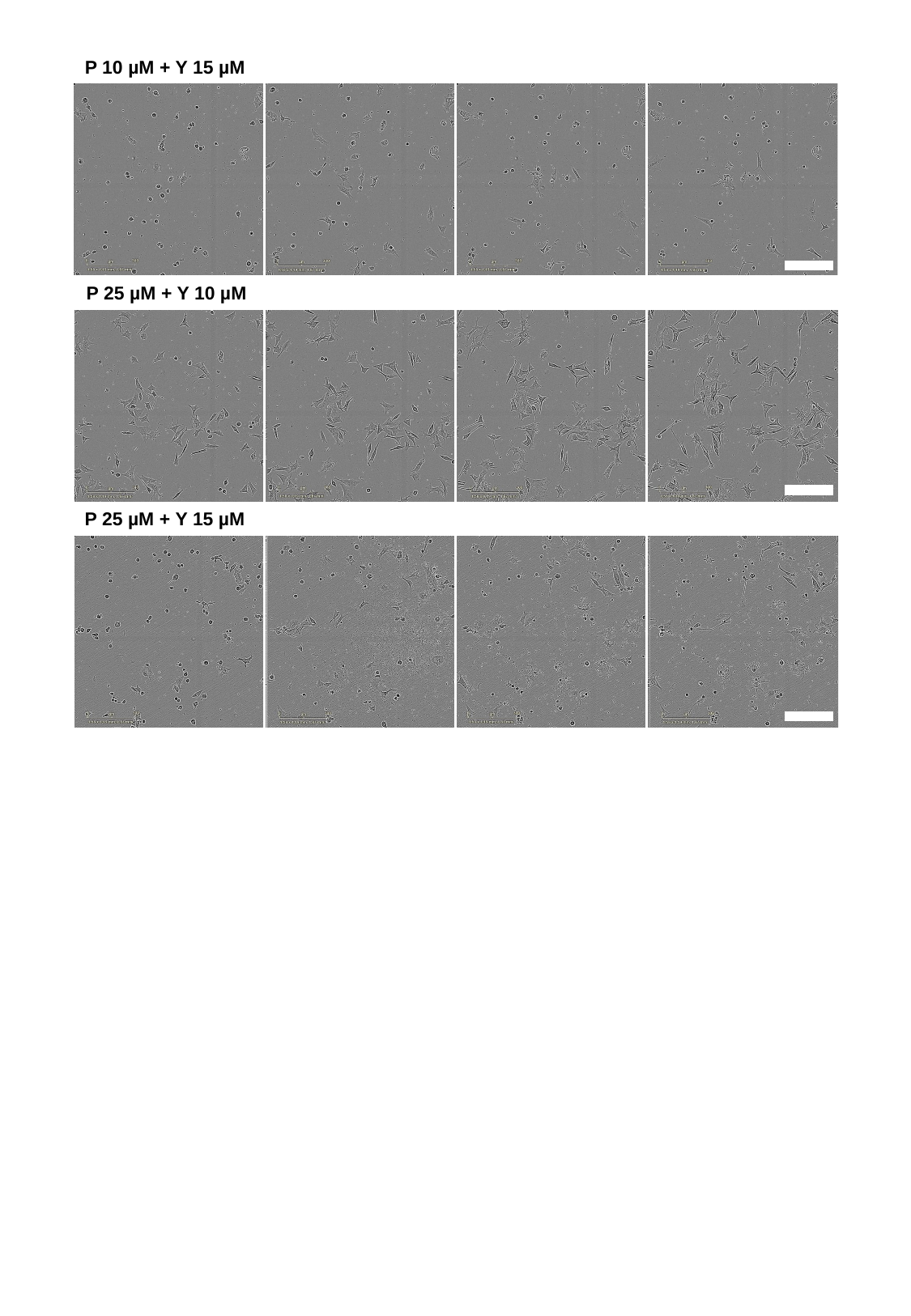

P 10 µM + Y 15 µM
P 25 µM + Y 10 µM
P 25 µM + Y 15 µM

## Slide 8
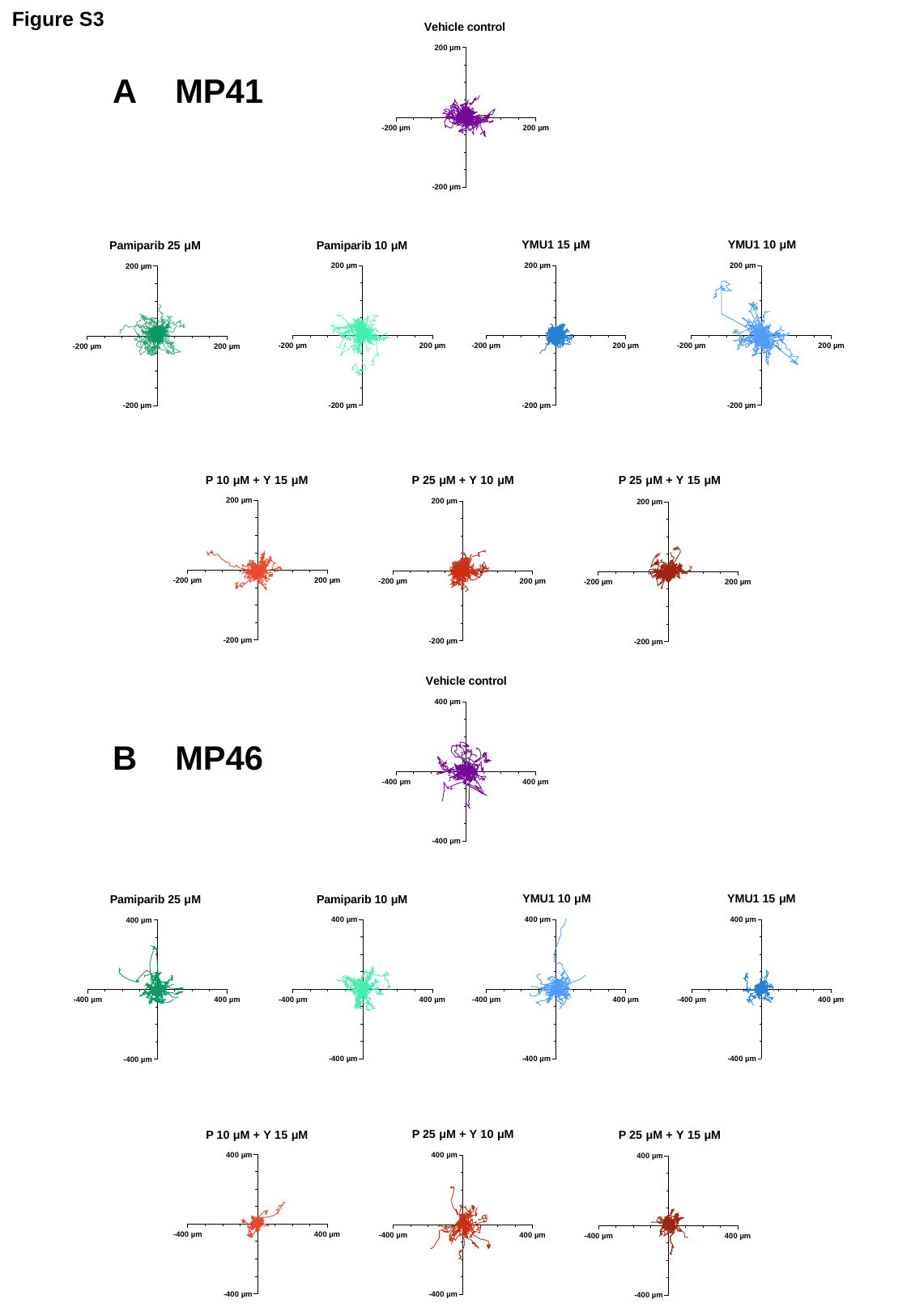

Figure S3
A
MP41
B
MP46
